# Treatment of genital lesions with diode laser vaporization

**DOI:** 10.1186/s12894-015-0033-6

**Published:** 2015-05-08

**Authors:** Mário Maciel de Lima, Mário Maciel de Lima, Fabiana Granja

**Affiliations:** Department of Urology, Coronel Mota Hospital, Rua Levindo Inácio de Oliveira, 1547, Paraviana, Boa Vista, RR CEP: 69307-272 Brazil; Biodiversity Research Center, Federal University of Roraima (CBio/UFRR), Boa Vista, Brazil

**Keywords:** Genital lesion, Laser treatment, Laser diode vaporization, Urology

## Abstract

**Background:**

Genital warts caused by human papillomavirus (HPV) infection are the most common sexually transmitted disease leading to anogential lesions. Although the laser therapy has been shown to be effective in a number of conditions, the use of laser diode vaporization in urological applications and the understanding on its effectiveness as a treatment for various urological conditions is limited. Therefore, the aim of this study was to evaluate the efficacy of diode laser vaporization as a treatment for genital lesions.

**Methods:**

Patients presenting with genital lesions at the urology outpatient clinic at Coronel Mota Hospital, between March 2008 and October 2014, were enrolled into the study. Data collected included age, gender, duration of the lesion, site of the lesion and numbers of the lesions, length of follow-up, recurrence of lesions after treatment and whether there were any complications.

**Results:**

A total of 92 patients were enrolled in the study; 92.4% (n = 85) male; mean age (± SD) 27.92 ± 8.272 years. The patients presented with a total of 296 lesions, with a median of 3 lesions each, including penis (n = 78), urethra (n = 4) lesions, and scrotum (n = 2) lesions. Lesions ranged in size from 0.1 to 0.5 cm^2^, most commonly 0.3 cm^2^ (n = 38; 41.3%), 0.4 cm^2^ (n = 21; 22.8%) or 0.5 cm^2^ (n = 20; 21.7%). Patients most commonly reported that they had their lesions for a duration of 12 (n = 29; 31.5%) or 6 months (n = 23; 25.0%). Eighteen patients (19.6%) had a recurrence after their 1^st^/conventional treatment. There were no incidences of post–operative infection or complications from the laser diode vaporization.

**Conclusions:**

Laser diode vaporization can be considered as an alternative method for treating genital lesions in urology, with satisfactory results in terms of pain, aesthetic and minimal recurrence.

## Background

Genital lesions are a relatively common condition which may be caused either by sexually transmitted human papillomavirus (HPV) infection, as a result of non–infectious inflammatory diseases such as psoriasis and lichen planus, by a drug reaction and also as premalignant lesions that can progress to carcinoma. Regardless of origin, genital lesions are both a source of considerable discomfort and a cause of embarrassment and psychological repercussions. Genital lesions can often persist for prolonged periods, frequently a number of years and can re–occur after treatment.

Genital warts caused by HPV are the most common sexually transmitted disease and a major cause of anogential lesions. In addition to physical and psychological implications of HPV warts, there is also a substantial economic cost, estimated at $6 billion annually in the United States [1]. No specific antiviral therapies are available to cure HPV anogenital warts; treatment therefore relies on removal of warts or limiting spread through anti-proliferative or immunomodulation therapy [2]. However recurrence rates can be high due to the widespread infection or subclinical lesions that are not identified at the time of treatment. The variety of different treatment options for genital warts can be loosely grouped into three categories: topical agents, systemic agents, and surgical therapies [2].

One surgical therapy that is showing increasing use across dermatological conditions is diode laser therapy. Diode lasers are semiconductors that change electrical energy into light energy through the use of solid‑state elements, such as aluminum and gallium. The light beam which is released by the diode laser falls within the visible and invisible range of near infrared waves (with wavelengths varying between 800 and 980 nm) and is able to vaporize soft tissue due to its high water content. These light beams are poorly absorbed by the hard tissue and therefore do not damage nearby hard tissue. By focussing the beam on the desired area for incision, a highly precise focal spot can be created. By adjusting the focus of the beam, the intensity of the laser light can be varied, which allows cauterization of small blood vessels and lymphatics to decrease post–operative swellings and sealing of nerve endings to reduce post-operative pain [3-5]. Studies suggest that side effects of diode laser therapy are generally mild [6-9]. However laser therapy can be expensive and is not widely available. Research to determine the efficacy of laser therapy for the treatment of different conditions is therefore important in order to justify investment in laser equipment and training in the use of laser therapy.

Laser therapy has been shown to be effective in a number of conditions, with probably the largest body of work conducted to examine the efficacy of laser surgery for the removal of different oral lesions such as simple soft tissue surgery (e.g. frenectomy, gingival contouring plasty) [3,10-12], vascular lesions (e.g. hemangiomas, telangiectasias) [4,5] and pigmented lesions [3]. Other areas where lasers have become a key option for treatment include cosmetic applications such as laser hair removal and laser tattoo removal, and various dermatological applications, including conditions such as syringoma, xanthelasma palpebrarum, recalcitrant warts, rhinophyma, epidermal nevi, condyloma and intraepithelial neoplasia and milia [13-19]. These studies have suggested a number of advantages of laser surgery over traditional scalpel surgical procedures, such as greater precision, a relatively bloodless surgical and postsurgical course, sterilization of the surgical area, minimal swelling and scarring, coagulation, vaporization, cutting, minimal or no suturing, and less or no postsurgical pain [12].

There have been few studies to date that have examined the efficacy of laser therapy as a destructive therapy for genital warts [20-23]. Because warts are vascular, laser therapy should result in instant coagulation and therefore provide bloodless removal of the lesion. The few studies of genital lesions conducted to date suggest clearance rates ranging between 23% and 52% for carbon dioxide and pulse dye laser therapy; however recurrence rates as high as 77% have also been reported [19,20,22]. However, the description of the use of laser diode vaporization in urological applications is still limited, and further work is required to fully understand the effectiveness of laser diode vaporization as a treatment for various urological conditions. The aim of this study was therefore to evaluate the efficacy of diode laser vaporization treatment in genital lesions.

## Methods

### Study population

This was a prospective study of patients presenting with genital lesions between March 2008 and October 2014. The study was conducted in the urology department outpatient clinic at Coronel Mota Hospital. Patients were eligible to be enrolled in the study if they presented with a genital lesion and were willing to provide informed written consent to the study. No other eligibility criteria were applied. The study was conducted under the ethical approval provided by ethical review board of Coronel Mota Hospital.

### Data collection

Following consent, a full demographic and medical history was taken for each participant. Data collected included age, gender, duration of the lesion, site of the lesion and number of the lesions, length of follow-up, recurrence of lesions after treatment and whether there were any complications.

### Diagnosis and treatment protocol

Upon presentation and written consent to participate in the study, patients underwent a physical examination to diagnose the lesion/s. No additional endoscopy was used at for diagnosis. Each patient then underwent laser diode vaporization of their lesion/s according to a standardized protocol. Firstly the warts lesion was prepared by sterilizing the area with povidone-iodine solution (10%) and infiltrating locally with 2% xylocaine hydrochloride without adrenalin. The laser light was then applied using either a circular or radial contact method. In all cases a Zap Laser, LLC® model Z2006AP was used, with a power density of 1.2 W/cm^2^ CW (continue wave). Laser treatment was generally applied for 5–15 seconds for each lesion. For patients with a larger number of lesions, multiple treatment sessions were used. For example, for a patient with 10 lesions, a total of 4 treatment sessions were used.

### Patient follow-up

After treatment, patients were seen at 7 days in order to check for early post-operative complications. They were then followed up at approximately 4 weeks intervals for 12 weeks. Any associated complications that occurred during this time, such as infection, scarring, hyperpigmentation, hypo- pigmentation or any other sequelae, were looked for and recorded if they occurred. Follow-up of patients continued at the end of the 12 weeks in order to collect data on potential longer term complications.

## Results

### Patient characteristics

A total of 92 patients were enrolled in the study, of which 7 (7.6%) were female and 85 (92.4%) were men (Table 1). All patients were treated with diode laser due to verrucoid lesions (<0.5 cm^2^) in genital and skin. The mean age of the study cohort was 27.92 ± 8.272, ranging from 15 to 53 years. Together, the participants had a total of 296 lesions; this included one patient who had more than 10 lesions. Most commonly patients had three (n = 28; 30.4%), two (n = 18; 19.6%), four (n = 14; 15.2%), or five 5 (n = 15; 16.3%) lesions, with the median number of lesions being three. The lesions ranged in size from 0.1 to 0.5 cm^2^, most commonly 0.3 cm^2^ (n = 38; 41.3%), 0.4 cm^2^ (n = 21; 22.8%) or 0.5 cm^2^ (n = 20; 21.7%). A total of 78 patients had penis lesions, whilst four had external urethral meatus lesions, two had scrotum lesions, one had an anal mucosa lesion, and there were seven participants who had lesions on their face or neck (four with lesions on the neck, two with oral lesions and one with lesions on their face). A further four patients had a frenulectomy and therefore no lesions were reported for these patients.

**Table 1:** **Patient characteristics**

**Characteristics**	**n (%)**
**Age (years)**	
15–19	12 (13.0)
20–29	45 (48.9)
30–39	27 (29.3)
40–49	7 (7.6)
50–59	1 (1.1)
**Gender**	
Male	85 (92.4)
Female	7 (7.6)
**Location of lesion**	
Penis	78
Urethra	4
Scrotum	2
Anus	1
Face	1
Neck	4
Oral	2
Frenulectomy	4
**Number of lesions**	
1	8 (8.7)
2	18 (19.6)
3	28 (30.4)
4	14 (15.2)
5	15 (16.3)
6	2 (2.2)
7	1 (1.1)
8	1 (1.1)
>10	1 (1.1)
**Size of largest lesion (cm** ^**2**^ **)**	
0.2	9 (9.8)
0.3	38 (41.3)
0.4	21 (22.8)
0.5	20 (21.7)
**Duration of lesions (months)**	
<6	15 (16.3)
6–11	27 (29.3)
12–23	30 (32.6)
24–35	14 (15.2)
36	2 (2.2)
**Recurrent after 1** ^**st**^ **/conventional treatment**	
Yes	18 (19.6)
No	70 (76.1)

Patients most commonly reported that they had had their lesions for a duration of 12 (n = 29; 31.5%) or 6 months (n = 23; 25.0%), with reported durations ranging from 1 to 36 months. A total of 18 patients (19.6%) had had a recurrence after their 1^st^/conventional treatment.

### Treatment outcomes

**Figure 1:**
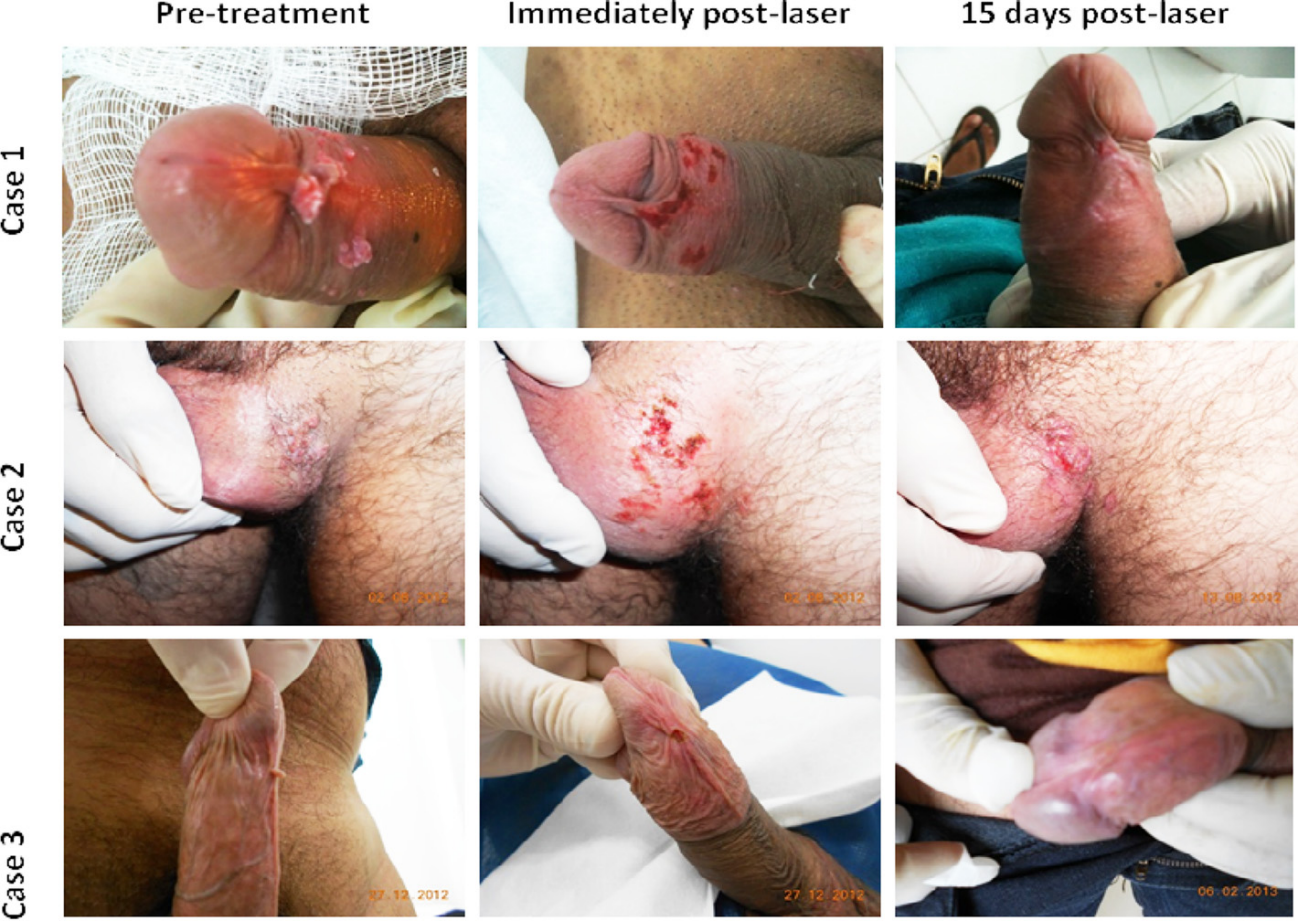
Representative images from the three genital lesion cases taken before the treatment, immediately after laser treatment and 15 days post-treatment.

Figure 1 shows examples from three cases of genital lesions before treatment, immediately post laser treatment and 15 days post laser treatment. Post-operative infection was not observed in any of the patients. Mild pain, oozing, oedema, and scales were observed during the first seven days after laser irradiation. Depending on the size of the lesions the healing process, which usually occurs by granulation tissue formation, took between 2–3 weeks. Patient follow up continued for between 2 months and 6 years (mean 33.90 ± 25.68 months) after the intensive follow-up period. No complications from the laser diode vaporization were observed, including no reported problems with urination or ejaculation and no strictures or scars.

## Discussion

In our study we have evaluated the effect of diode laser vaporization for the treatment of genital warts. The treatment protocol used in our study is comparable to that used in the previous small studies examining pulsed dye laser therapy for the treatment of genital warts [22]. We found only a small incidence of recurrence after laser diode treatment. Recurrence rates in previous studies have varied largely, with one study reporting a recurrence rate of 12.6% at one month post-laser therapy, whilst others have reported recurrence rates as high as 77% using carbon dioxide laser and pulsed dye laser [19,20,22]. Variation in reported recurrence rates may partially reflect the varying follow-up times within the individual studies. Some minimal discomfort and oedema was observed during the immediate period post–laser treatment, as expected and in line with findings in previous studies [24]. Notably, we also observed no long-term complications in any of the patients enrolled in the study, which is in line with previous reports of the excellent safety profile of laser therapy as a treatment for genital warts [9,19,22]. Importantly our study supports reports in the literature that laser treatment decreases post-operative pain by sealing nerve endings and reduces blood loss by cauterizing blood vessels [12].

We found no evidence of infection in any of the patients within our study. This may be expected since laser beams have a natural sterilization effect, causing the evaporation of bacteria, viruses and fungi within the immediate vicinity of the beam and therefore decreasing the possibility of local infection.

## Conclusion

Our study suggests that laser diode vaporization provides a good option for the treatment of genital lesions. Laser diode vaporization appears to provide a good clearance rate for the elimination of the verrucae, whilst at the same time having a low complication and side–effect profile, in terms of scarring and post-operative pain, and a low incidence of recurrence. Therefore laser diode vaporization can be considered as an alternative method for treating genital lesions in urology, with satisfactory results in terms of pain, aesthetic and minimal recurrence.

## Consent

Written informed consent was obtained from the patient for the publication of this report and any accompanying images.
